# Genotype-Dependent Variation of Nutritional Quality-Related Traits in Quinoa Seeds

**DOI:** 10.3390/plants10102128

**Published:** 2021-10-07

**Authors:** Sara Granado-Rodríguez, Susana Vilariño-Rodríguez, Isaac Maestro-Gaitán, Javier Matías, María José Rodríguez, Patricia Calvo, Verónica Cruz, Luis Bolaños, María Reguera

**Affiliations:** 1 Departamento de Biología, Campus de Cantoblanco, c/Darwin 2, Universidad Autónoma de Madrid, 28049 Madrid, Spain; sara.granado@uam.es (S.G.-R.); isaac.maestro@uam.es (I.M.-G.); luis.bolarios@uam.es (L.B.); 2 Vitrosur Lab SLU, Algodonera del Sur, Carretera Trebujena C-441 (km 5.5), Lebrija, 41740 Sevilla, Spain; svilarino@algosur.com; 3 Agrarian Research Institute “La Orden-Valdesequera” of Extremadura (CICYTEX), 06187 Badajoz, Spain; javier.matias@juntaex.es (J.M.); veronica.cruz@juntaex.es (V.C.); 4 Technological Institute of Food and Agriculture of Extremadura (CICYTEX), 06007 Badajoz, Spain; mariajose.rodriguezg@juntaex.es (M.J.R.); patricia.calvo@juntaex.es (P.C.)

**Keywords:** quinoa, genotype, nutritional traits, seed quality

## Abstract

Exploiting the relationship between the nutritional properties of seeds and the genetic background constitutes an essential analysis, which contributes to broadening our knowledge regarding the control of the nutritional quality of seeds or any other edible plant structure. This is an important aspect when aiming at improving the nutritional characteristics of crops, including those of *Chenopodium quinoa* Willd. (quinoa), which has the potential to contribute to food security worldwide. Previous works have already described changes in the nutritional properties of quinoa seeds due to the influence of the environment, the genotype, or their interaction. However, there is an important limitation in the analyses carried out, including the outcomes that can be translated into agronomical practices and their effect on seed quality. In the present study, several seed nutritional-related parameters were analyzed in 15 quinoa cultivars grown in a particular environmental context. Important agronomical and nutritional differences were found among cultivars, such as variations in mineral or protein contents and seed viability. More importantly, our analyses revealed key correlations between seed quality-related traits in some cultivars, including those that relate yield and antioxidants or yield and the germination rate. These results highlight the importance of considering the genotypic variation in quinoa when selecting improved quinoa varieties with the best nutritional characteristics for new cultivation environments.

## 1. Introduction

Quinoa (*Chenopodium quinoa* Willd.) is a halophytic crop that belongs to the *Amaranthaceae* family. It can be adapted to a wide variety of agroecosystems and is resistant to stressful environmental conditions [1,2]. Moreover, quinoa possess excellent nutritional properties [1,3]. All these characteristics have resulted in a global expansion of quinoa cultivation during the past three decades [4], and consequently, this explains why quinoa can be found from the harsh climatic conditions associated with high altitudes of the Andean Altiplano (reaching over 3500 m above sea level) to coastal areas. 

Quinoa was first domesticated by pre-Columbian cultures more than 7000 years ago, when it became one of their main sources of nutrients, given the lack of animal protein. After the Spanish conquest, quinoa was highly rejected but maintained by indigenous farmers, despite the introduction of Old-World species. These farmers domesticated the cultivars, preserving the genetic diversity found currently in quinoa [5]. This genetic diversity can be divided into different ecotypes that include thousands of accessions (16,422) [6] that reflect the diffusion from the center of origin of the crop around Lake Titicaca [5]. 

Currently, quinoa is still the principal protein source in many areas of the Altiplano. The nutritional value of quinoa seeds was rediscovered during the last decades of the 20th century, leading to a renewal of its production [7]. This led to a rapid spread in its cultivation, from very few countries around the Andean Altiplano in the 1980s to 123 countries in 2018 [4]. The success in its international acceptance has been possible in part due to the nutritional characteristics of the seeds. Quinoa seeds are considered pseudocereals because they resemble cereal grains in their high starch content and overall morphology. However, quinoa seeds are gluten-free and have a low glycemic index, being low in sugar and calories. They present a high protein content with an excellent balance of essential amino acids, as well as high contents of fiber, lipids, carbohydrates, minerals, and bioactive compounds, such as vitamins (B2 and E), carotene, tocopherols, and other molecules with antioxidant properties, like flavonoids and other phenolic compounds [8,9,10,11,12]. 

Antioxidants are of economic interest since they can minimize rancidity and increase the shelf-life of food products [13]. Moreover, they are also of nutritional interest due to their health-related benefits. Antioxidants have been found to reduce the risks of cancer and cardiovascular disease and to present anti-inflammatory and anti-microbial activity [14,15]. Quinoa also shows unique fiber, lipid (with a high ratio of omega-6:omega-3), and micro- and macronutrient profiles (often higher than cereal-based products) that give quinoa seeds beneficial characteristics, such as decreasing the risk of cancer and cardiovascular and inflammatory diseases; decreasing blood pressure, diabetes, and development of hemorrhoids; and weight control [16], thus improving intestinal health [9]. Overall, quinoa seeds provide nutritional and health benefits, which is why quinoa is considered a “superfood of the future” [17,18].

Furthermore, quinoa is offered as a nutritious food for low-income countries and constitutes a crop able to grow on marginal lands (including those with limited rainfalls or poor soil quality) not suitable for other major crops [8]. This brings interesting opportunities for the agriculture of low-income countries and, generally, for those countries where agricultural water supply is (or will be soon) limited. These include Mediterranean countries where there is an urgent need to develop sustainable practices to mitigate the impacts of climate change and human pressure on soil resources [8]. This is especially relevant within the current climate change and food security context [19]. Besides, it should be noted that quinoa is not only consumed by humans, as its different plant parts can be used as a nutritionally valuable forage crop, apt for feeding sheep, pigs, cattle, poultry, and horses [20]. 

Importantly, it should be noted that quinoa exhibits a strong variability in cultivar-specific responses to environmental variation. There are reports of different environmental conditions impacting some seed quality-related parameters in quinoa, including seed size and protein or mineral contents, depending on the specific genotype [21,22,23,24,25]. Thus, different cultivars of quinoa have shown substantial differences in the nutritional characteristics, which also vary with the environment. However, it is still unclear if the parameters that were evaluated are stable among cultivars at different locations or if steady correlations can be found between nutritional-related parameters. A recent work by Granado-Rodriguez et al. [26] showed that some quinoa cultivars, Titicaca and Vikinga, present better quality-related traits (including higher protein contents), despite not being the most productive cultivar when growing in the Northwestern part of Spain. In line with this, selecting the best adapted genotypes for a particular cultivation environment is key, in terms of yield potential and biotic and abiotic stress tolerance but also considering the different nutritional traits [27]. Thus, we need to better understand the genetic and environmental factors determining the nutritional characteristics of quinoa. This will be achieved through the use of conventional and molecular tools that will help to unlock the rich biodiversity and cultivation potential of this crop [28].

Therefore, in the present study, we aimed at analyzing differences in seed quality among the 15 quinoa genotypes examined. We analyzed a variety of nutritional-related parameters with cultivation potential in the Southern region of Spain. Differences in the parameters analyzed were found among varieties for most of the parameters analyzed, including plant height, panicle length, mildew incidence, lodging, seed weight and area, protein content, germination rates, seed viability, mineral contents (except for Mg), antioxidants, and saponin contents. Furthermore, important correlations between different seed quality-related parameters dependent on the genotype (including yield and antioxidants or germination rates) support the presence of genetic determinants of nutritional quality in quinoa.

## 2. Results

### 2.1. Plant Performance and Physiological and Agronomical Traits 

In this study, we aimed at analyzing different nutritional traits under filed conditions. Seeds of 15 different quinoa cultivars were sown on 27th January 2018, and plants were harvested on either July 18th or August 1st (Supplementary Figure S1). Cultivars ‘A-SE-03’, ‘A-SE-06’, ‘A-SE-07’, ‘A-SE-09’, ‘A-SE-12’, ‘A-SE-13’, and ‘A-SE-15’ were harvested in July (*Harvesting 1,* Supplementary Figure S1), while cultivars ‘A-SE-01’, ‘A-SE-02’, ‘A-SE-04’, ‘A-SE-05’, ‘A-SE-08’, ‘A-SE-10’, ‘A-SE-11’, and ‘A-SE-14’ presented longer life cycles, and they were harvested later (*Harvesting 2,* Supplementary Figure S1). Total seed yield varied among cultivars, with A-SE-08 cv. Being the cultivar that presented the highest seed yield (4.7 t/ha), followed by A-SE-11 cv. and A-SE-05 cv. (with 3.5 and 3.4 t/ha, respectively). A-SE-01 was the cultivar with the lowest seed yield (0.96 t/ha). Precipitation across the growing season was concentrated in the first months of cultivation (February to April), coinciding with plant nascence and emergence (Supplementary Figure S1), while scarce precipitation was registered from flowering to harvesting time (May–July). Daily mean temperatures varied from 8.7 °C (at sowing) to 25.7 °C and 30.7°C (at harvesting of short- and long-life-cycle cultivars, respectively), and they increased progressively along the growing season (Supplementary Figure S1). Inflorescences started appearing in May. At the seed maturation stage, temperatures higher than 25°C were registered for all cultivars.

**Figure 1:**
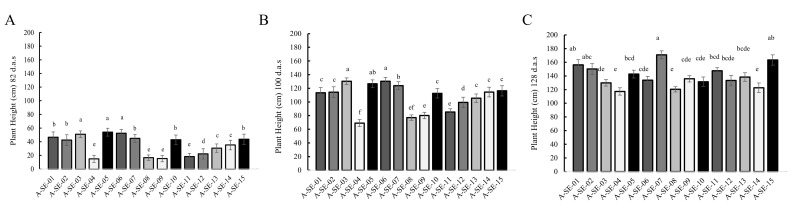
Plant height. Plant height (cm) was determined in the 15 cultivars analyzed at (**A**) 82, (**B**) 100, and (**C**) 128 d.a.s. Error bars represent the standard deviation. Bars that do not share the same letters show statistically significant differences following the Kruskal–Wallis test at a *p*-value < 0.05 for 82 d.a.s. and 100 d.a.s. and the ANOVA test and Tukey post-hoc test at a *p*-value < 0.05 for 128 d.a.s. (days after sowing).

Plant height also showed significant variations among cultivars (Figure 1). At early stages (82 days after sowing (d.a.s)), A-SE-05 cv., A-SE-03 cv., and A-SE-06 cv. plants were the tallest, with plant lengths of 54 ± 5.76 cm, 51 ± 4.79 cm, and 52.4 ± 5.6 cm, respectively, and, at middle stages (100 d.a.s.), A-SE-03 cv. and A-SE-06 cv. plants were still the tallest, with heights of 130.4 + 8.94 cm and 130.4 ± 6.33 cm, respectively. At the latest stages (128 d.a.s), A-SE-07 cv. and A-SE-15 cv. were the tallest plants, presenting as 170.90 ± 22.87 cm and 163.40 ± 31.99 cm in height, respectively. On the other hand, A-SE-04 cv. was the shortest cultivar throughout the season, going from 14.9 ± 5.27 cm tall at the early stage, to 69.10 ± 11.74 cm tall at the middle stage, and to 117.30 ± 25.12 cm at the end of the life cycle. In line with this parameter, lodging was also evaluated in this study. Thus, it was observed that the cultivars A-SE-07 and A-SE-03 presented greater lodging resistance (3% of lodging plants), while the cultivar A-SE-12 showed great sensitivity to lodging, with 36% of affected plants (Supplementary Figure S2). 

**Figure 2:**
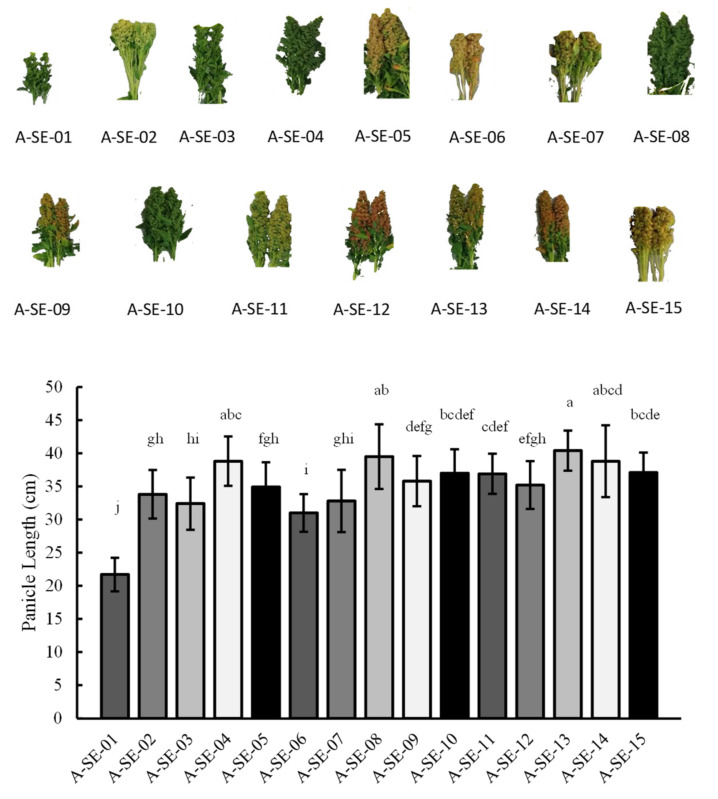
Panicle lenght. Panicle length (cm) was determined in the 15 cultivars analyzed. Error bars represent the standard deviation. Bars that do not share the same letters show statistically significant differences following the Kruskal–Wallis test at a *p*-value < 0.05.

Panicle length was determined in the different cultivars, evaluated at 128 d.a.s. Most of the cultivars showed panicle lengths between 30 cm and 40 cm, with A-SE-01 cv. having the lowest values (21.70 ± 2.53 cm) and A-SE-13 cv. showing the highest (40.40 ± 3.03 cm) (Figure 2). In addition to the panicle length, the weight of those panicles was also measured (Supplementary Figure S2). The results indicated that having larger panicles usually correlated with heavier weights, although some exceptions were observed (i.e., A-SE-02 cv. was 33.8 ± 3.67 cm long but presented the biggest weight of 3.20 kg/25 panicles). Interestingly, lodging resistance was not related to panicle length, as the cultivars with contrasting lodging resistance (A-SE-07, A-SE-03, and A-SE-12) did not show significant differences in panicle lengths.

**Figure 3:**
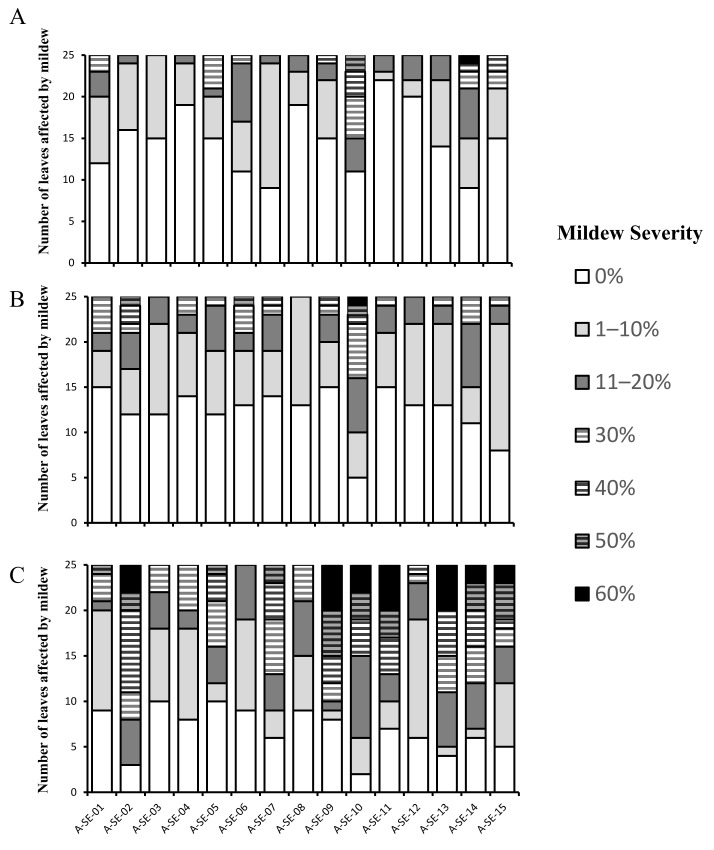
Mildew incidence and severity. Mildew incidence and severity were determined as described in the Methods section. Mildew severity degreewas expressed as the percentage (%) of leaf affected by the pathogen (leaf area converage of 0%, 1–10%, 11–20%, 30, 40%, 50%, or more than 60%). Mildew incidence and severity were evaluated at different developmental stages: at (**A**) 82 days after sowing (d.a.s.) (upper panel), (**B**) 100 d.a.s. (middle panel), and (**C**) 128 d.a.s (bottom panel).

Mildew incidence and severity were analyzed throughout the experiment (at 82, 100, and 128 d.a.s.) (Figure 3). A-SE-03 cv. and A-SE-08 cv. were the less-affected cultivars at the early stages (82 and 100 d. a. s.), and A-SE-06 cv. and A-SE-12 cv. were less affected at the later stages (128 d. a. s.). Meanwhile, A-SE-09 cv., A-SE-10 cv., and A-SE-11 cv. were the most afflicted ones in terms of severity, especially at 128 d.a.s.

**Figure 4:**
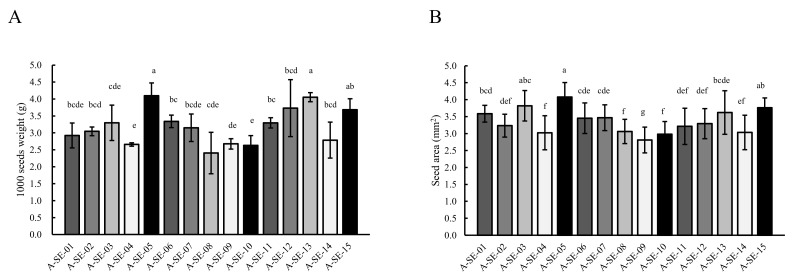
Seed weight and seed area. (**A**) Seed weight (g) and (**B**) area (mm^2^) were determined among the different cultivars studied. Error bars represent the standard deviation. Bars that do not Scheme 0.

Seed weight exhibited an effect that was related to the cultivar (Figure 4A). The cultivars A-SE-05 cv., A-SE-13 cv., and A-SE-15 cv. presented the heaviest seeds, while A-SE-04 cv., A-SE-10 cv., and A-SE-09 cv. showed the lightest seed weights. 

Seed area showed a high correlation with seed weight (Supplementary Figure S3). A-SE-05 cv., A-SE-03 cv., and A-SE-15 cv. presented the largest seeds, while A-SE-04 cv., A-SE-08 cv., A-SE-09 cv., and A-SE-10 cv. had the narrowest seed areas (Figure 4B).

### 2.2. Germination Rates and Seed Viability

**Figure 5:**
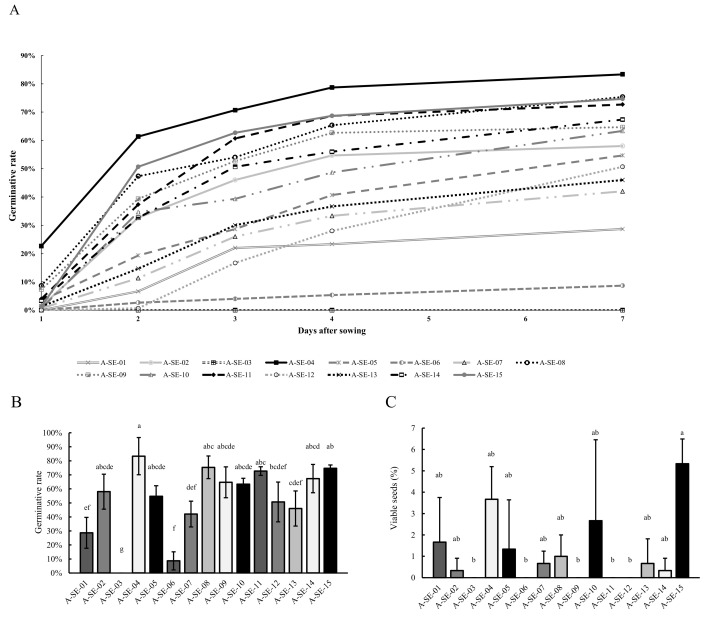
Germination rates (%) and seed viability. (**A**) Time course of the germination percentage (%) of quinoa seeds 1, 2, 3, 4, 5, 6, and 7 seven days after sowing (d.a.s.); (**B**) germination rate percentage (%) 7 d.a.s.; and (**C**) percentage (%) of viable seeds. Error bars represent the standard deviation. Bars that do not share the same letters show statistically significant differences following a Kruskal–Wallis test by ranks for multiple comparisons at a *p*-value < 0.05.

Germination rates were determined from the seeds harvested to evaluate the germination capacity (Figure 5A). Noticeably, differences were found in the germination rates of the various cultivars analyzed. A-SE-04 cv. and A-SE-15 cv. showed germination rates above 50% at 3 d.a.s, and A-SE-04 cv. reached 80% germination rate at 7 d.a.s (Figure 5B). Meanwhile, A-SE-03 seeds were unable to germinate, and A-SE-06 cv. and A-SE-01 cv. did not overtake 20% germination rates at 3 d.a.s. On the other hand, even though A-SE-12 cv. seeds showed a germination delay, they were able to reach a germination rate of almost 50% at 7 d.a.s, being close to the A-SE-05 cv. seeds at 7 d.a.s. 

Seed viability was determined to complete the physiological analysis of the seeds (Figure 5C). For most of the cultivars, except for A-SE-15 cv., A-SE-04 cv., A-SE-03 cv., and A-SE-06 cv., seed viability showed no correlation with seed germination, as it was generally severely reduced in most of the seeds tested.

### 2.3. Protein Content

**Figure 6:**
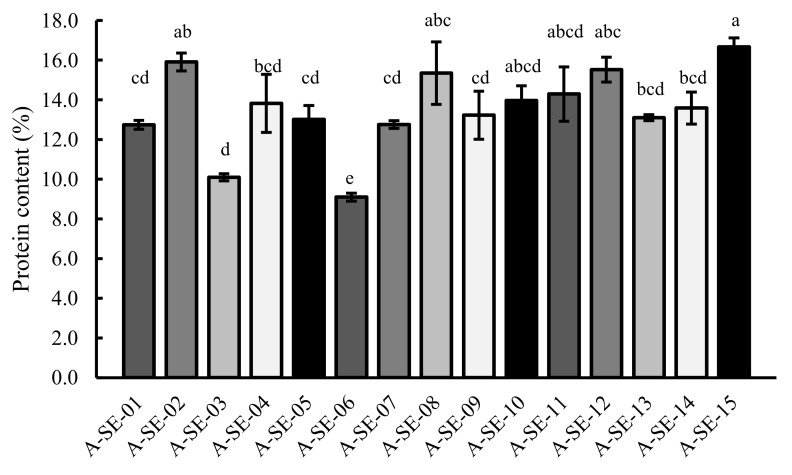
Seed protein content (%). Protein content was determined in seeds of the 15 cultivars evaluated. Error bars represent the standard deviation. Bars that do not share the same letters show statistically significant differences following the Kruskal–Wallis test by ranks for multiple comparisons at a *p*-value < 0.05.

Total protein contents in seeds revealed variations among cultivars (Figure 6). The cultivars A-SE-15 cv. and A-SE-02 cv. showed the highest contents, while, in contrast, A-SE-06 cv. and A-SE-03 cv. showed the lowest. Interestingly, as shown in Supplementary Figure S3, protein content positively correlated with TPC, FRAP, saponin content, Zn, Mg, and P and negatively with C/N ratio and Na content. Regarding the agronomical parameters, protein content correlated negatively with panicle height and panicle biomass and positively with the germination rate (Supplementary Figure S3).

### 2.4. Mineral Content

The total contents (as %) of phosphorous (P), potassium (K), calcium (Ca), magnesium (Mg), (and as mg/Kg) sodium (Na), copper (Cu), iron (Fe), manganese (Mn), and zinc (Zn) in quinoa seeds were determined to analyze the effect of the genotype on this nutritional-related parameter (Table 1). Some mineral nutrients, such as Mg, did not show significant variation among genotypes or, as in the case of K, showed a small fluctuation. On the contrary, minerals such as Zn showed a steeper variation. A-SE-12 cv., A-SE-15 cv., and A-SE-13 cv. showed the highest Zn contents, while A-SE-03 cv. and A-SE-09 cv. presented the lowest. Among cultivars, it should be noted that A-SE-12 cv. presented higher contents of P, Ca, Fe, and Zn and intermediate levels of the rest of minerals, while A-SE-15 cv. presented the highest contents of P, Cu, and Zn and the lowest of Ca, Na, and Fe. At the same time, A-SE-03 cv., A-SE-04 cv., and A-SE-06 cv. had higher contents of Ca and Na and lower contents of P and Cu.

### 2.5. Antioxidant Capacity

**Figure 7:**
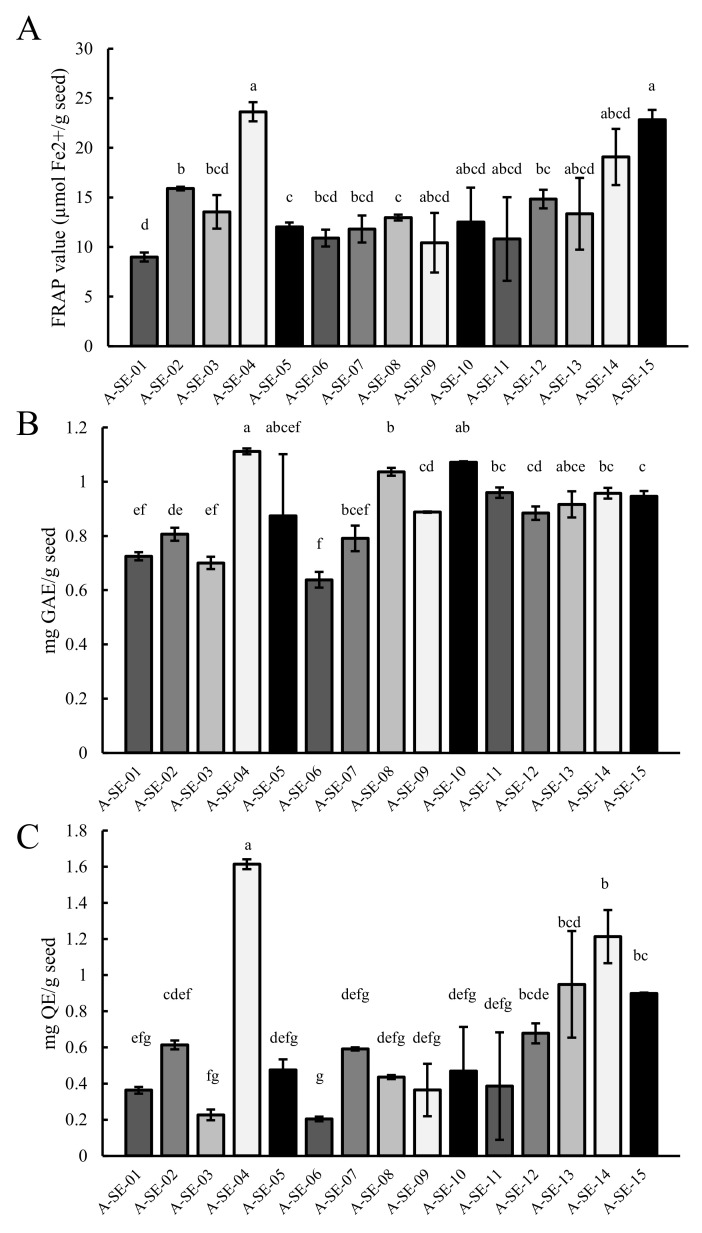
Antioxidant capacity of quinoa seeds. (**A**) Antioxidant power, determined by an FRAP assay, is expressed as µmol of Fe^2+^ per gram of seed. Statistical differences were analyzed through a Welch’s ANOVA test, followed by a Games–Howell post-hoc test. (**B**) TPC is expressed as milligrams of gallic acid equivalents (GAE) per gram of seeds. The statistical analysis performed was a Welch’s ANOVA test, followed by a Games–Howell post-hoc test. (**C**) TFC is expressed as milligrams of quercetin equivalents (QE) per gram of seeds. A Kruskal–Wallis test by ranks was performed for multiple comparisons. Bars that do not share the same letters show statistically significant differences at a *p*-value < 0.05. Error bars represent the standard deviation.

We evaluated the antioxidant capacity of the seeds by performing an FRAP assay and a quantification of total polyphenols (TPC) and flavonoids (TFC) contents (Figure 7). Among cultivars, A-SE-04 cv. showed the highest antioxidant capacity and phenolic and flavonoid contents, followed by A-SE-15 cv. On the contrary, A-SE-01 cv., together with A-SE-06 cv., showed the lowest values. The rest of cultivars showed distinct patterns, presenting changes among the antioxidant-related parameters here evaluated. For instance, A-SE-10 cv. showed intermediate and high FRAP and TFC values, respectively, and low TPC levels. 

### 2.6. Saponin Content

**Figure 8:**
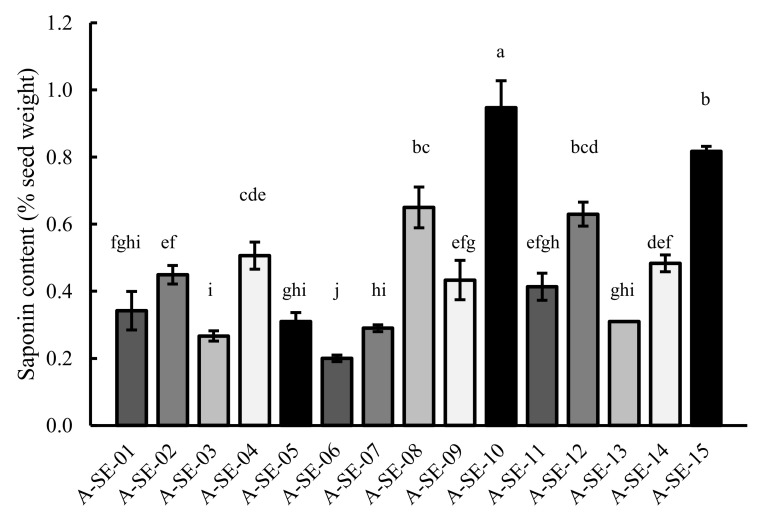
Saponin content. Saponin content was determined in seeds of the 15 cultivars evaluated. Error bars represent the standard deviation. Bars that do not share the same letters show statistically significant differences following the Kruskal–Wallis test by ranks at a *p*-value < 0.05.

Saponin content was quantified in the cultivars studied (Figure 8). A-SE-06 was the cultivar showing the lowest saponin content, while A-SE-10 was the cultivar with the highest saponin level. All of the cultivars exceed the limit of 0.11%, established to classify quinoa varieties as sweet [29]; however, none of them presented a content higher than 1%, which is usually overtaken by bitter quinoa seeds [30]. 

### 2.7. Principal Components Analysis (PCA)

**Figure 9:**
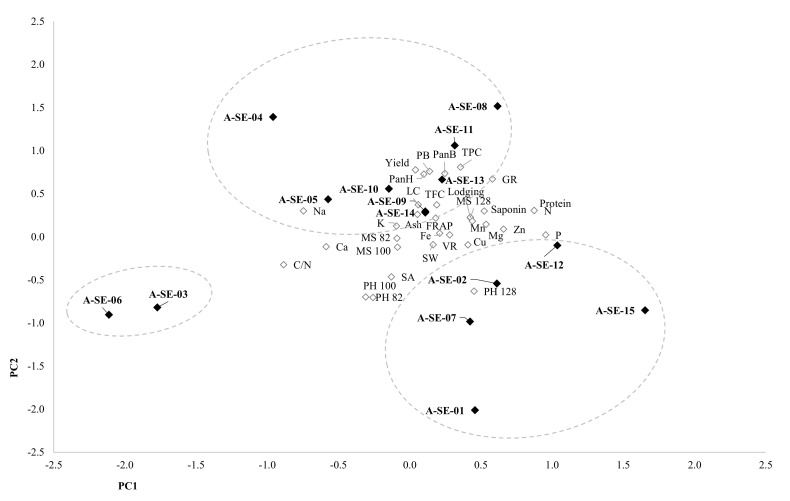
Principal components analysis (PCA). Biplot of main components 1 and 2 for the cultivars sown and for the variables tested. Component 1 (X-axis) was contributed mainly by protein and saponin contents, germination rate, minerals (P, Mg, Ca (-), Na (-), Mn, Cu, Zn), lodging, plant height at 128 d.a.s., and mildew at 128 d.a.s. Component 2 (Y-axis) included germination rate, total phenolics content, plant height (-), plant biomass, panicle height, and panicle weight (-).

A Pearson’s correlation coefficient test was performed to analyze the correlation between variables (Supplementary Figure S3) and a principal component analysis (PCA) to reduce the number of variables. This analysis identified five principal components that were able to explain 74.76% of the variance. Component 1, which contributed to 21.31% of the variance, was mainly explained by the protein and saponin contents and most minerals’ contents (P, Ca, Mg, Mn, Cu, and Zn contributed positively, and Ca and Na contributed negatively) and by the germination rate, lodging, plant height, and mildew severity at 128 d.a.s. For this new variable, A-SE-12 cv. and A-SE-15 cv. showed high values, while A-SE-03 cv. and A-SE-06 cv. presented the lowest (Figure 9). There were correlations between most of these variables, but those between protein content and germination rate (r = 0.801), protein and P contents (r = 0.846), P and Zn contents (r = 0.728), and protein and saponin contents (r = 0.695) were the strongest (Supplementary Figure S3). Component 2 contributed to the variance with 18.30%, and it comprised panicle length and biomass, plant dry weight, yield, germinative rate of seeds, and total phenolic content, and inversely, it comprised plant height (at three time-points) and seed area. Plant height at early stages (82 and 100 d.a.s.) correlated negatively with the final plant biomass, yield, germinative rate of seeds, and protein and phenolic contents, while the panicle height and plant weight at 128 d.a.s. correlated positively with these parameters. Strong correlations were found between the phenolic content and the germinative rate and panicle height (r = 0.884 and r = 0.780, respectively) (Supplementary Figure S3). For this component, there were high values in A-SE-08 cv. and A-SE-04 cv. and low values in A-SE-01 cv (Figure 9). Component 3 explained 12.19% of the variance and comprised the viability rate, flavonoid contents, and antioxidant capacity, and inversely, it comprised ash, K, and Fe contents. Both viability and germinative rate correlated with each other and with the antioxidant capacity and phenolic and flavonoid contents. There was also a strong correlation between ash and K content (r = 0.851) since K is the main mineral present in quinoa (Table 1). A-SE-04 cv. and A-SE-15 cv. showed high component 3 values, and A-SE-09 cv., A-SE-11 cv., and A-SE-13 showed low values. Area and seed weight and K and Cu contents contributed positively to component 4 (explaining 11.96% of variance), and Fe and Ca contents contributed negatively. Area and seed weight showed a strong correlation (r = 0.748). A-SE-13 cv. presented the highest, and A-SE-01 cv., A-SE-09 cv., and A-SE-10 cv. presented the lowest values for component 4. Component 5 (11.00% of variance) comprised saponin content in seeds, panicle length, and mildew severity at three stages. There was a correlation between mildew severity at 82 and 100 d.a.s. but not with severity at 128 d.a.s. Saponin content and panicle height also showed a strong correlation (r = 0.655). A-SE-10 cv. showed high component 5 values, while A-SE-12 cv. showed the lowest. Life cycle duration correlated with yield, germination rate of seeds, and their phenolic contents.

Plotting component 1 against component 2 revealed three clusters of cultivars (Figure 9). The first cluster was made up of A-SE-03 and A-SE-06, and it was low for both components 1 and 2, which means they had taller plants at early stages but low yields; germinative capacity; and protein, P, saponin, and phenolic contents. These cultivars were also low for component 3, and they showed the lowest viability rate and low antioxidant capacity but a high ash content. The second cluster, comprising A-SE-01 cv., A-SE-02 cv., A-SE-07 cv., A-SE-12 cv., and A-SE-15 cv., had low component 2 values, especially for A-SE-01 cv., so they were tall plants with smaller panicles and lower yields but higher component 1 values than the first cluster, so generally, they had higher protein, P, and Cu contents and lower Ca and Na contents than A-SE-03 cv. and A-SE-06 cv. A-SE-05 cv., A-SE-08 cv., A-SE-09 cv., A-SE-10 cv., A-SE-11 cv., A-SE-13 cv., and A-SE-14 cv. comprised the third cluster, which had high component 2 values, having shorter plants and larger panicles, heavier plants, higher yields and germination rates, and higher protein and phenolic contents. A-SE-04 cv. and A-SE-15 cv. showed the highest component 3 values, with the largest viability rates and FRAP values, but A-SE-04 cv. had lower component 1 values, with high Ca and Na contents and lower Cu and Zn contents. A-SE-15 cv. had high component 1 values, and it had high germination rates and protein, P, Cu, and Zn contents and low Ca and Na contents.

A path analysis was also performed to define the direct and indirect contributions of each trait to seed yield (Supplementary Tables S1 and S2). First, a predictive multiple linear regression model was performed following the stepwise method in order to find traits with a direct effect on germination rates (Supplementary Table S1) and yield (Supplementary Table S2). As shown in Table S2, germination rates would be affected positively by the phenols (TPC) and P contents and indirectly by physiological or agronomical parameters, such as seed area or panicle biomass, or by biochemical properties of seeds, such as protein or saponin content. On the other hand, yield would be explained in a negative way by the seed weight and panicle height and positively by the panicle biomass and total biomass, meaning that these parameters may directly impact the seed yield of the quinoa varieties here analyzed.

## 3. Discussion

Quinoa is often compared to cereals and even considered a ‘pseudocereal’ due to the similarities in the composition and uses of their seeds [31]. It presents unique nutritional properties that make its cultivation very interesting [32]. However, its full yield potential is not yet reached for new cultivation areas, with levels similar to those of cereals such as wheat or rice before the Green Revolution [2]. The center of origin of quinoa is the Andean Altiplano [33], but in the last decades, quinoa has been introduced as an alternative emerging crop in more than 75 countries [5]. Along with its expansion, it has been observed that the establishment and adaptation of quinoa cultivars to these new agroclimatic contexts can result in changes in the nutritional properties of quinoa seeds, which are associated with variations in the genotype (G), the environment (E), and their interaction (GXE) [14,21]. Thus, there is still much left for researchers and breeders to do in order to develop quinoa cultivars that are better adapted to specific locations that present high yields while also maintaining or even improving the nutritional value of the seeds. In this study, we evaluated physiological and agronomical characteristics together with different nutritional-related traits of seeds harvested from 15 different quinoa cultivars grown in southern Spain, aiming to expand our knowledge of the relationship between yield and the nutritional quality of quinoa seeds and therefore, contribute to the selection of quinoa cultivars more appropriate for cultivation in a particular area of interest.

As previously described, through the PCA, we could classify the cultivars into three clusters, depending on their distinct characteristics (Figure 9). At earlier stages of development (82 and 100 d.a.s.), plants from clusters 1 (A-SE-03 cv. and A-SE-06 cv.) and 2 (A-SE-01, A-SE-02 cv., A-SE-07 cv., and A-SE-15 cv.) were the tallest, but only those from cluster 2 remained taller at a later developmental stage (128 d.a.s., beginning of grain maturation) (Figure 1). Cluster 1 and cluster 2 plants also presented lower panicle lengths and biomass (Figure 2) and smaller seed yields. Seeds from these cultivars did not show higher nor lower seed weights or areas, and there was no correlation between these variables and yield (Supplementary Figure S3). From this, it can be assumed that plants from clusters 1 and 2 invested more resources towards growing at earlier stages of development but they invested fewer resources in the development of the panicle and seed biomass. It should be noted that, for these plants, lower yields were not correlated with reduced seed weights, but with smaller panicles producing fewer seeds. This negative relation between plant height and seed yield and positive relation between panicle size (biomass and height) and yield (Table S2, Supplementary Figure S3) have been previously described [34,35,36]. Furthermore, Gómez et al. [37] reported a correlation between plant height and yield in quinoa. They postulated that quinoa’s low yield can be explained by its low sink capacity and that an increase in reproductive partitioning, reducing plant height, could positively impact yield in this crop, as had happened previously to wheat and rice during the Green Revolution [38]. Furthermore, some reports have found a positive correlation between yield and plant height, pointing as well to the influence of the environment in controlling this trait. Therefore, the implication of the environment should be investigated for the varieties here analyzed [39]. Nonetheless, plant height can be an important trait for breeding. Thus, further research analyzing endogenous factors that may control quinoa height and its relationship with yield and lodging (i.e., phytohormones) should be considered in quinoa. 

Intriguingly, previous works have observed correlations between yield and seed nutritional-related traits, like the positive correlation found between yield and the antioxidant capacity or the K content or the negative correlation between yield and protein content or the amount of different amino acids [22,26,40]. However, these studies compared the nutritional profiles of quinoa seeds harvested from different cultivars but grown in different environmental conditions (with variations in the sowing date, the cultivation location, and/or the year of cultivation). Thus, the variations on the nutritional traits of the seeds in these cases were mainly determined by differences in the environmental conditions at the seed-filling stage, which affect both yield and seed-quality traits [26]. In the present study, only the genetic factor was evaluated, so the lack of correlations between yield and seed nutritional-related traits suggests that there might be no link between them. Therefore, those relations are only relevant when introducing the cultivars to new environments where they are not yet adapted.

Downey mildew, caused by the fungus *Peronospora variabilis* Gäum, is one of the main diseases affecting quinoa on a global scale [41]. Optimal conditions for mildew development are found at high humidity (>80% RH) and moderate temperatures (between 18 °C and 22 °C), but its expansion can be interrupted by long periods of sunny and dry conditions [41]. In this study, high RH was found in March, when most of the precipitation occurred and plants were still emerging or developing their first true leaves (Supplementary Figure S1). However, temperatures at that time were lower than 18 °C, which is suboptimal for mildew development. Mildew produces chlorotic patches on leaves, which may result in premature defoliation by the plant as a defense mechanism. This reduction of the photosynthetic area can lead to an atrophied development and smaller panicles, which in turn, lowers seed yield [42]. When the infection occurs at early stages of development of quinoa, 20–40% yield penalties have been estimated for mildew-resistant cultivars [43], and losses of up to 99% have been estimated in susceptible cultivars [42]. However, the impact of mildew on well-established mature plants is less important than abiotic stresses [44], since the defoliation caused by the disease and by the natural senescence of the plant overlap [41]. In the present study, mildew incidence and severity were limited at early stages (82 and 100 d.a.s.), with severities lower than 10% in most cultivars (Figure 3), despite not using plant protectants. However, severity increased at a later stage (128 d.a.s.), with A-SE-02 cv. (cluster 2), A-SE-09 cv., A-SE-10 cv., A-SE-11 cv., and A-SE-13 cv. (cluster 3) being the most affected ones and A-SE-01 cv., A-SE-12 cv. (cluster 2), A-SE-03 cv., A-SE-06 (cluster 1), A-SE-04 cv., and A-SE-08 cv. (cluster 3) being the least affected (Figure 3). Mildew severity did not correlate to yield nor to any seed nutritional traits (Supplementary Figure S3) [44], which suggests that the cultivars tested were resistant to mildew and did not suffer significant yield losses related to this disease. 

Saponins are secondary metabolites found in the pericarp of quinoa seeds, which cause a bitter taste when they are present in substantial amounts. They also have a negative effect on the bioavailability of minerals like Fe and Zn [45]. For these two reasons, saponins are considered as “anti-nutrients”. Different breeding programs have been focused on the development of cultivars with very low seed saponin contents (sweet quinoa cultivars) [28]. Koziol [46] established the limit between sweet seeds and bitter seeds at 0.11% of seed weight, while Mastebroek et al. [47] considered those with saponin contents between 0.02% and 0.04% as sweet seeds and those with contents between 0.47% and 1.13% as bitter seeds. All of our samples fitted the definition of a bitter seed by Koziol [46], but only A-SE-04, A-SE-08, A-SE-10 cv. (cluster 3), A-SE-12 cv., and A-SE-15 cv. (cluster 2) would be considered bitter following the Mastebroek et al. [47] criterion, and all samples would be ‘low-saponin’ seeds according to Medina-Meza et al. [30]. In this regard, it should be noted that sweet varieties are normally preferred, since the elimination process of saponins is avoided. However, some farmers prefer bitter cultivars, because saponins may confer resistance to biotic stresses [28]. Although saponins have been hypothesized to also give mildew resistance to quinoa [48], since they possess antifungal activities [49], no correlation has been found between seed saponin contents and mildew resistance in quinoa [50] (Supplementary Figure S3).

Saponin is a highly genotype-dependent seed trait in quinoa [51], and no correlation to other seed traits had been found previously. In the present study, we found, for the first time to our knowledge, a correlation between saponin content a other seed quality-related traits, like germination, protein content, and flavonoid content (Supplementary Figure S3) [52,53,54].

Germination capacity is an important seed characteristic for breeding programs. This is because any genetic potential achieved through breeding efforts cannot be exploited if seed establishment in the field is not successful. In this study, most cultivars surpassed the 50% germination rate, but A-SE-13, A-SE-07, A-SE-01, and especially A-SE-03 and A-SE-06 (cluster 1), showed very low germination rates (Figure 5 and Figure 9). Interestingly, a correlation between seed germination rates and the panicle’s characteristics and seed yield of the mother plants was found [26] (Supplementary Figure S3), but germination rates were also influenced by nutritional traits of seeds. Both the correlation and pathway analyses showed a strong effect of the phenolic compounds and the P contents on the germination capacity of seeds (Supplementary Figure S3, Table S1). These results are in part supported by previous works, as a positive correlation between phenolic compounds and the germination capacity of quinoa seeds has previously been found [26]. Furthermore, a stimulating effect of these compounds on the germination capacity has been reported as well in the close quinoa relative species *Chenopodium album* L. [55]. On the other hand, P is present in quinoa seeds, mainly as phytate [56], a form of P storage not bioavailable for many monogastric animals, including humans [57,58]. During germination, however, the phytase activity catalyzes the hydrolysis of the phytate [59], providing inorganic phosphate essential for the metabolism of the seed at the beginning of germination [60,61]. According to Nadeem et al., [62], a higher phytate content also means more hydrolysis and thus, higher phosphate available during germination. This may explain the correlation between P content and germination (Supplementary Figure S3, Table S1), since higher P contents in seeds are related to faster germination and better establishment of seeds in the field [63]. A strong positive correlation was also found between the germination capacity and protein content (Supplementary Figure S3), probably associated with the role that storage proteins play in germination [64]. Noteworthily, the environmental conditions may differently impact the cultivars included in this study and, consequently, result in variations in seed germination [25,65]. To further explore this aspect, the cultivation in consecutive years should be considered in future works.

In the present study, the protein content of seeds varied depending on the cultivar, with values ranging between 12.7% and 16.7% with the exception of two cultivars, the low-performing A-SE-03 and A-SE-06 (cluster 1). These two cultivars presented seed protein contents of 9–10%, closer to the values found in cereals like maize and barley and lower than the values found in wheat [66]. The contents of the rest of the cultivars fell within the range expected for quinoa seeds, with A-SE-02, A-SE-12, A-SE-15 (cluster 2), and A-SE-08 (cluster 3) exceeding 15% [66]. However, it should be noted that the importance of the quinoa protein does not only rely on the quantity, but also on the quality. Quinoa seed proteins contain all amino acids, and they are present in a proper balance, similar to the complete amino acid profile found in cow’s milk and close to the ideal equilibrium recommended by the FAO for human consumption [7,67].

The ash content ranged from 3.03% to 3.73%, depending on the cultivar, although few significant differences were found among cultivars (Table 1). These were normal values for quinoa but generally higher than those of cereals like wheat or rice [66,67]. The minerals that were present in higher amounts were K, P, Ca, and Mg, while Na, Fe, Zn, Mn, and Cu contents were the lowest (Table 1) [67]. All these minerals fell within the ranges previously reported for quinoa seeds [26,66], and some of them, like K, Ca, Mg, and Na, were higher than those found in cereals like maize, barley, rice, and wheat [7]. The high contents of Fe, Ca, and Mg are especially important, since they are minerals less present in gluten-free products, and thus, quinoa seeds can be an important source of these minerals for people with coeliac disease [67].

Noteworthily, the contents were significantly different among cultivars for all minerals except for Mg (Table 1). The variations in mineral contents in quinoa seeds had previously been reported to be cultivar-dependent, but they also respond to environmental differences during plant growth [22,26]. For instance, the cultivar A-SE-03 showed high Ca and Na and low P and Zn contents, while A-SE15 cv. showed high P, Cu, and Zn contents and low Ca, Na, and Fe contents (Table 1). P content was high, but according to Konishi et al. [56], P is mostly found in quinoa seeds as phytic acid, which can form complexes with Fe, Zn, Mg, and Ca, reducing their bioavailability for human digestion [68]. Interestingly, Ruales and Nair [45] noted that, in feeding experiments with rats, there were no differences in Fe availability in quinoa-supplemented diets compared to those supplemented with FeSO_4_. Thus, further evaluation of the actual effect of quinoa seeds’ phytic acid on Fe, Zn, Mg, and Ca availability should be conducted in order to elucidate which percentage of these minerals’ contents is actually taken up during human digestion and if these contents reach the human nutritional requirements [69].

A correlation between phenolic compounds and flavonoids contents and the antioxidant capacity was expected (Supplementary Figure S3) [26,70]. The antioxidant capacity and phenolic compounds content are genotype-dependent in quinoa seeds [71], although they can also change depending on the environmental context [21,26,72]. In the present study, the antioxidant capacity, TPC, and TFC were comparable to those found in previous studies (Figure 7) [15,21,26,71] and changed depending on the cultivar. For instance, the cultivars A-SE-04 and A-SE-15 and A-SE-04, A-SE-08, and A-SE-10 showed the highest levels of antioxidant capacity and TPC, respectively, while A-SE-01 presented the lowest antioxidant capacity, and A-SE-03 and A-SE-06 exhibited the lowest TPC and TFC (Figure 7). These results correlated well with other seed-related traits, like protein content and germination rates, and with seed yield (Supplementary Figure S3). This, together with the overall health benefits of antioxidants, make TPC and TFC interesting traits for quinoa breeding programs.

Targeting phenotypic traits such as physiological, agronomical, or seed nutritional-related parameters might be very useful when aiming at performing phenotyping screenings or for breeding programs. The selection of the best quinoa cultivars for production can be based on the results obtained for some of the parameters here discussed (including the results of their correlation). For instance, the present work highlights that the cultivar A-SE-08 is the most promising one amongst the 15 cultivars studied, based on the higher protein contents, yield, germination rate, and P and phenolic contents. Targeting these traits can be very useful for selecting the best adapted varieties for a particular area of cultivation.

## 4. Materials and Methods

### 4.1. Plant Material, Experimental Design, and Location

Field trials were conducted in a field experimental station located in Lebrija (Seville, Spain, 36.88° N, 6.13° W) in clay-loam soil. Sowing to harvesting dates took place from January to August of 2018. Fifteen different quinoa cultivars, given by Algosur S. A. (Lebrija, Spain), were used in this study, encoded as follows: ‘A-SE-01’, ‘A-SE-02’, ‘A-SE-03’, ‘A-SE-04’, ‘A-SE-05’, ‘A-SE-06’, ‘A-SE-07’, ‘A-SE-08’, ‘A-SE-09’, ‘A-SE-10’, ‘A-SE-11’, ‘A-SE-12’, ‘A-SE-13’, and ‘A-SE-14’. (Supplementary Figure S4).

Each cultivar was sown on January 27th in two replicates of non-randomized plots, with dimensions of 4.5 m × 266 m, spacing between rows of 0.75 m, and 0.02 m within rows. The plot dimension was large enough to ensure uniformity according to our field experiments previously performed. A drilling machine was used to sow with a density of seeds of 2 kg·ha^−1^.

During the experiment, different measurements of agronomical traits were taken. Plant height and downy mildew incidence and severity were measured at 82, 100, and 128 d.a.s, which corresponded to different developmental stages: fully emerged plants, panicle emergence, and beginning of seed ripening, respectively. Furthermore, at 128 d.a.s., the panicle length and weight of 25 plants per cultivar were measured. Plant harvesting took place when plants had naturally dried out at different time points: July 18th (172 days after sowing) for the cultivars ‘A-SE-03’ cv. ‘A-SE-06’ cv., ‘A-SE-07’ cv., ‘A-SE-09’ cv., ‘A-SE-12’ cv., ‘A-SE-13’ cv., and ‘A-SE-15’ cv. and on August 1st (186 d.a.s.) for ‘A-SE-01’ cv., ‘A-SE-02’ cv., ‘A-SE-04’ cv., ‘A-SE-05’ cv., ‘A-SE-08’ cv., ‘A-SE-10’ cv., ‘A-SE-11’ cv., and ‘A-SE-14’ cv. Total seed yield was quantified from an 11.25 m^2^ plot for each cultivar, and the dry weight of 20 plants was measured.

Climatological data, including total precipitation, relative humidity (RH), and temperature, were obtained daily from a local climatological station (Supplementary Figure S1). Sprinkler irrigation was supplemented at different developmental stages: at seed sowing (30 L/m^2^), 5 days after sowing (30 L/m^2^), at the beginning of branching (30 L/m^2^), at flowering (50 L/m^2^), and during grain filling (50 L/m^2^).

### 4.2. Seed Weight and Area

Seeds were manually counted and weighed in an analytical balance. The seed area was analyzed using the open-source software ImageJ (http://rsbweb.nih.gov/ij/ accessed on 12 November 2020). Images were taken using an Olympus SZ61 stereomicroscope (Olympus Corporation, Shinjuku, Tokyo, Japan) and processed with the AnalySIS GetIT image software (analysis getIT 5.1, Olympus Corporation, Shinjuku, Tokyo, Japan). To determine seed weight, 1000 seeds were used per replication, and 3 replications were utilized. For seed area measurement, 50 seeds were used per replication, and 3 replications were utilized

### 4.3. Seed Germination Rate

Quinoa seeds (using 50 seeds per replication and 3 replications per cultivar) were sterilized by soaking first in ethanol 70% for two minutes, next in bleach 50% with a droplet of Tween-20 for two minutes, and then rinsing several times in distilled water (H_2_O). Sterilized seeds were sown on a double layer of filter paper, wet with distilled water, on Petri dishes and then transferred to a growth chamber under darkness and a controlled temperature of 23 °C. The germination rate was counted daily for the first week after sowing. Seeds were considered as germinated when the radicle protrusion was longer than 2 mm.

### 4.4. Seed Viability

Seed viability tests were performed using the tetrazolium method (2,3,5-triphenyl-2H-tetrazolium chloride). First, seeds (using 100 seeds per replication and 3 replications per cultivar) were imbibed in distilled water at 30 °C for an hour to facilitate longitudinal and superficial cuts of the embryo and to ensure a homogeneous dying of the seed tissues. After cutting, seeds were submerged in 1% tetrazolium chloride at 30 °C for two hours. Seeds with more than 50% staining in the embryonic tissue were considered viable.

### 4.5. Saponin Content

To determine saponin content, 20 mL of 50 % ethanol was added to 1 g of powdered sample and left to macerate for 72 h at room temperature. Then, the extracts were filtered into 20 mL volumetric flasks. The samples were then filtered using a 0.45 μm nylon Filter-Lab syringe and analyzed by High-Performance Liquid Chromatography Fluorescence and Diodo Array Detection (HPLCDAD, Serie 1100, Agilent Technologies, Waldbronn, Germany) at 225 nm [73]. Saponin (Merck, Germany) was used as a standard. The results were expressed in g saponin 100 g^−1^ of fresh weight.

### 4.6. Protein Content

The protein content was determined according to AOAC Official Methods [74], using an elemental analyzer Leco TruSpec (LECO TruSpec (LECO, MI, USA)) and considering a conversion factor of 6.25 [75].

### 4.7. Mineral Content

The mineral content was analyzed following the official methods of analysis of the Spanish Ministry of Agriculture [76]. Phosphorus content was determined using a spectrophotometer UV-VIS (Hitachi U-2810, Tokyo, Japan) (yellow coloration, 430 nm). Potassium was determined through flame atomic emission spectroscopy. Calcium, magnesium, sodium, iron, copper, manganese, and zinc contents were assessed using flame atomic absorption spectroscopy (AAS) (SpectrAA 110, Agilent Technologies Inc., Palo Alto, CA, USA) after mineralizing the samples with H_2_O and HCl (35%).

### 4.8. Ferric Reducing Antioxidant Power (FRAP) Assay

To obtain total extracts, seeds were ground to a fine powder, and 100 mg of the flour was homogenized in 1 mL of an extraction buffer, consisting of methanol (50%), acetic acid (1%), and distilled water (49%). These samples were vortexed for 2 min and kept in the dark at 4 °C for 48 h before centrifugation for 15 min at 13500 rpm. The supernatants were stored at −20 °C until their use in the FRAP and flavonoid content assays.

The antioxidant capacity of seeds was determined following the procedure described by Benzie and Strain [77]. The FRAP reagent consisted of a mix of 300 mM acetate buffer (pH 3.6), with 10 mM TPTZ in 40 mM HCl and 20 mM FeCl_3_·6H_2_O at a ratio of 10:1:1 (*v*/*v*/*v*). A total of 20 µL of the sample extract and 180 µL of the FRAP reagent were added into a 96-well microplate and incubated for 4 min. Absorbance was read at 593 nm using a microplate reader Lector Multi-ModalSynergy HTX (BioTek Instruments, Inc., Winooski, VT, USA). The antioxidant capacity was calculated from a calibration curve obtained with iron (II) sulfate (FeSO_4_). The FRAP value was expressed as µmol of Fe^2+^ g^−1^ of seed.

### 4.9. Total Phenol Content (TPC)

Extracts were obtained after homogenizing 100 mg of seed flour in 1 mL of ice-cold methanol (95%). The mix was vortexed, and centrifuged at 13,500 rpm for 5 min after 48 h kept in the dark at 4 °C.

The content of polyphenols was measured following the protocol described by Ainsworth and Gillespie [78]. Briefly, 100 µL of the sample extract or standard were added to 200 µL of the Folin–Ciocalteu reagent (10%) and the mixture was vortexed for 1 min. Next, 800 µL of sodium carbonate (7.5%) were added. The mix was then incubated for 2 h in the dark. The samples were centrifuged in order to eliminate precipitates. Absorbance was read at 765 nm using a microplate reader Lector Multi-ModalSynergy HTX (BioTek Instruments, Inc., Winooski, VT, USA). Concentrations of gallic acid between 20 µg·mL^−1^ and 200 µg·mL^−1^ in methanol (95%) were used as a standard, and thus, the TPC was expressed as mg of gallic acid equivalents per grams of quinoa seed (mg GAE·g^−1^). 

### 4.10. Total Flavonoid Content (TFC)

Flavonoid content was determined following the procedure described by Valenzuela [79]. The same extracts as in the FRAP assay were used. Briefly, 30 µL of the sample extract or standard, 10 µL of aluminum chloride (AlCl_3_) 10%, 10 µL of sodium acetate (NaC_2_H_3_O_2_) 1 M, and 250 µL of dH2O were mixed and incubated for 30 minutes. The absorbance was read at 415 nm using a microplate reader Lector Multi-ModalSynergy HTX (BioTek Instruments, Inc., Winooski, VT, USA). Quercetin dissolved in ethanol (80%) was used as a standard, with concentrations ranging from 10 µg·mL^−1^ to 140 µg·mL^−1^. The results were expressed in mg of quercetin equivalents per gram of quinoa seed (mg QE·g^−1^).

### 4.11. Statistical Analysis

To analyze the differences between cultivars, different one-way ANOVA tests were performed. For variables where normality and equal variances could be assumed, a one-way ANOVA test was performed, followed by a Tukey post-hoc test to perform multiple comparisons at a probability level of 5% (*p* < 0.05). A one-way ANOVA on ranks (Kruskal–Wallis test by ranks) was performed when data did not present a normal distribution, and a Welch’s ANOVA test followed by a Games–Howell post-hoc test was performed when variances were not equal, both at a probability level of 5% (*p* < 0.05). Normality and equality of variances of the data were tested through a Kolmogorov–Smirnov’s test and a Levene’s test, respectively. A principal component analysis (PCA) was performed for plant parameters, like plant height at three stages, panicle length and biomass, plant biomass, mildew severity at different stages, resistance to lodging, life-cycle length, and yield, and for seed parameters, like viability and germination rates, 1000 seeds’ weight, seed area, saponin content, N and protein content and C-N ratio, FRAP value, phenols and flavonoids contents, and mineral contents. Correlations amongst variables were evaluated with a Pearson’s correlation coefficient test (Supplementary Figure S3). A sequential path analysis was performed to evaluate the specific contribution of different traits to yield or germination rate (Supplementary Tables S1 and S2). This analysis allowed ordering different variables as predictors of yield of the first, second, or third-order [80]. For this purpose, a stepwise multiple linear regression procedure was used, where variables that showed a weak contribution (*p* > 0.05) to the dependent variable (yield or germination rate) or high multicollinearity were automatically dropped from the model. The variables entered into the model were considered as first-order predictors, and the procedure was repeated using these variables as the response variable to identify traits that function as second-order predictors of yield. The tolerance and variance inflation factor (VIF) were used to measure the level of multicollinearity for each predictor trait. Tolerance lower than 0.1 or VIF values higher than 10 were considered as high levels of collinearity. Tolerance (1- R2i, where R2i is the coefficient of determination for the prediction of variable i by the predictor variables) is the amount of variance of the selected independent variable not explained by other independent variables. VIF (1/Tolerance) indicates the extent of the effects of other independent variables on the variability of the selected independent variable. The SPSS Statistics for Windows (Version 24.0., IBM Corp., Armonk, NY, USA) package was used for the statistical analyses.

## 5. Conclusions

This study revealed differences among cultivars for each physiological, agronomical, and seed nutritional-related trait analyzed, although there were similarities among some cultivars. For instance, A-SE-03 and A-SE-06, which clustered together in the PCA, showed taller plants at early stages of development but shorter plants with smaller panicles and lower yields at maturity (Figure 1 and Figure 9, Supplementary Figure S3). Regarding seed traits, these cultivars presented lower germination rates and lower protein, P, phenols, flavonoids, and saponins contents (Figure 5, Figure 7, Figure 8 and Figure 9, Table 1). On the contrary, the most promising cultivars for this agroclimatic context were those included in cluster 3, due to the higher yields, germination rates, and TPC (Figure 9). Since quinoa seeds are well known to be an excellent source of high-quality protein of non-animal origin, this is one of the main traits that makes quinoa a crop with a high nutritional quality, important for achieving food security locally and globally [81]. In this study, the higher protein contents were shown by A-SE-15 cv., A-SE-02 cv., A-SE-12 cv., and A-SE-08 cv. Therefore, overall, the cultivar A-SE-08 (cluster 3) can be considered the most promising cultivar for this particular area, since it not only presented higher protein contents, but also larger germination rates and P and phenolic contents. Having all these traits positively correlated can greatly facilitate the development of a better adapted cultivar. However, it should be noted that saponin content was also higher in this cultivar (Figure 9). Considering that reducing the saponin contents can improve nutritional quality and flavor [28,82], it would be interesting to explore the possibilities offered by agronomical management practices that allow the reduction of saponins [54,83].

Therefore, the results here presented highlight the importance of considering the genotypic variation in quinoa when selecting improved quinoa varieties with better nutritional characteristics for new cultivation environments. Further studies are required to determine which exact parameters are genotype-variable and which ones show genotypic stability.

## Figures and Tables

**Table 1:** Mineral seed contents. Mean ± SD mineral contents are presented as a percentage of seed weight (P, K, Ca, and Mg) or as mg/Kg (Na, Fe, Cu, Mn, and Zn). Statistical analysis following a Kruskal–Wallis test by ranks (P, K, Mg, Na, Mn, Cu, and Zn content) or a Welch’s ANOVA with a Games–Howell post-hoc test (Ca and Fe contents) were performed. Different letters under each mineral content show statistically significant differences between samples.

	Ash (%)	Nitrogen (%)	C/N ratio	P (%)	K (%)	Ca (%)	Mg (%)	Na (ppm)	Fe (ppm)	Mn (ppm)	Cu (ppm)	Zn (ppm)
**A-SE-01**	3.48 ± 0.02	2.04 ± 0.04	18.45 ± 0.35	0.29 ± 0.00	1.14 ± 0.01	0.30 ± 0.02	0.21 ± 0.00	146.98 ± 5.99	40.41 ± 0.27	19.57 ± 0.30	9.30 ± 1.42	29.18 ± 0.34
	b	cd	bc	bc	ab	abc	-	bcdefg	b	bcdef	bcde	efg
**A-SE-02**	3.22 ± 0.10	2.55 ± 0.07	14.81 ± 0.56	0.27 ± 0.00	1.03 ± 0.01	0.28 ± 0.01	0.19 ± 0.01	116.63 ± 7.66	38.98 ± 1.72	11.69 ± 0.05	11.70 ± 1.73	29.56 ± 0.44
	abc	ab	de	bc	b	bc	-	gh	b	f	bc	ef
**A-SE-03**	3.47 ± 0.07	1.62 ± 0.03	22.59 ± 0.28	0.17 ± 0.00	1.26 ± 0.01	0.38 ± 0.02	0.19 ± 0.00	233.42 ± 55.08	36.05 ± 1.35	16.65 ± 0.99	7.54 ± 0.56	23.93 ± 0.38
	abc	d	b	d	ab	a	-	ab	b	cdef	cdef	i
**A-SE-04**	3.03 ± 0.33	2.21 ± 0.23	17.47 ± 1.56	0.22 ± 0.02	0.98 ± 0.12	0.35 ± 0.02	0.20 ± 0.02	279.7 ± 37.63	32.85 ± 3.45	13.83 ± 0.95	7.86 ± 1.21	29.95 ± 0.52
	abc	bcd	bcd	cd	ab	ab	-	a	bc	ef	bcdef	def
**A-SE-05**	3.66 ± 0.27	2.08 ± 0.11	18.21 ± 0.92	0.27 ± 0.02	1.24 ± 0.10	0.35 ± 0.04	0.18 ± 0.01	173.49 ± 21.92	29.73 ± 2.74	13.42 ± 0.72	9.34 ± 0.80	31.60 ± 0.74
	abc	cd	bcd	bcd	ab	abcd	-	abcdef	bc	def	bcde	bcd
**A-SE-06**	3.11 ± 0.17	1.46 ± 0.03	25.34 ± 0.44	0.15 ± 0.00	1.13 ± 0.03	0.36 ± 0.12	0.17 ± 0.02	194.52 ± 59.36	35.08 ± 15.60	14.32 ± 6.60	9.40 ± 3.17	25.53 ± 1.23
	abc	e	a	e	ab	abcdef	-	abcde	bc	cdef	bcdef	ghi
**A-SE-07**	3.18 ± 0.09	2.04 ± 0.03	18.40 ± 0.45	0.26 ± 0.01	1.10 ± 0.02	0.18 ± 0.02	0.19 ± 0.00	125.43 ± 14.68	42.71 ± 3.77	19.92 ± 0.18	10.28 ± 0.31	30.85 ± 0.22
	bc	cd	bc	bcd	ab	def	-	efgh	abc	bcde	bc	cde
**A-SE-08**	3.43 ± 0.36	2.46 ± 0.25	15.65 ± 1.65	0.28 ± 0.03	1.15 ± 0.12	0.21 ± 0.02	0.22 ± 0.02	141.79 ± 12.98	43.18 ± 3.60	22.90 ± 0.38	10.32 ± 0.44	31.28 ± 0.80
	abc	abc	bcde	bc	ab	cde	-	cdefgh	abc	ab	bcd	bcd
**A-SE-09**	3.50 ± 0.47	2.12 ± 0.19	17.90 ± 1.50	0.26 ± 0.03	1.16 ± 0.16	0.27 ± 0.01	0.18 ± 0.02	153.07 ± 32.93	39.54 ± 4.68	22.19 ± 1.50	7.08 ± 0.53	23.80 ± 0.25
	abc	cd	bcd	bcd	ab	bcde	-	bcdefgh	abc	ab	def	i
**A-SE-10**	3.68 ± 0.34	2.24 ± 0.12	16.95 ± 0.99	0.24 ± 0.04	1.22 ± 0.17	0.25 ± 0.04	0.19 ± 0.03	191.24 ± 21.87	47.12 ± 8.56	20.77 ± 2.11	6.41 ± 1.01	25.32 ± 1.36
	abc	abcd	bcde	bcd	ab	abcdef	-	abcd	abc	abcd	ef	hi
**A-SE-11**	3.62 ± 0.63	2.29 ± 0.22	16.74 ± 1.57	0.28 ± 0.03	1.23 ± 0.21	0.24 ± 0.03	0.21 ± 0.03	194.76 ± 22.11	39.56 ± 6.24	20.05 ± 1.63	6.55 ± 0.68	28.76 ± 0.30
	abc	abcd	bcde	bcd	ab	abcdef	-	abc	abc	abcd	f	fgh
**A-SE-12**	3.26 ± 0.03	2.48 ± 0.10	15.13 ± 0.59	0.31 ± 0.01	1.12 ± 0.01	0.36 ± 0.01	0.19 ± 0.00	139.22 ± 16.03	56.39 ± 1.56	17.62 ± 0.51	8.98 ± 0.76	35.23 ± 0.21
	c	abc	cde	b	ab	a	-	defgh	a	cdef	bcdef	a
**A-SE-13**	3.73 ± 0.03	2.10 ± 0.02	17.93 ± 0.28	0.27 ± 0.01	1.39 ± 0.03	0.10 ± 0.03	0.18 ± 0.01	121.64 ± 15.62	35.17 ± 3.55	22.17 ± 1.15	19.52 ± 0.60	32.45 ± 0.58
	a	bcd	bcd	bcd	a	ef	-	fgh	bc	abc	a	bc
**A-SE-14**	3.34 ± 0.21	2.17 ± 0.13	17.52 ± 1.00	0.25 ± 0.01	1.16 ± 0.06	0.10 ± 0.02	0.20 ± 0.01	125.35 ± 15.11	33.19 ± 2.41	23.16 ± 1.16	18.46 ± 0.88	29.37 ± 0.97
	abc	bcd	bcd	bcd	ab	f	-	fgh	bc	a	ab	efg
**A-SE-15**	3.19 ± 0.08	2.67 ± 0.07	14.39 ± 0.48	0.35 ± 0.01	1.12 ± 0.02	0.09 ± 0.01	0.21 ± 0.01	100.87 ± 8.96	25.10 ± 1.29	20.34 ± 0.50	19.75 ± 0.96	34.09 ± 0.81
	bc	a	e	a	ab	f	-	h	c	abcd	a	b

## Data Availability

The data that support the findings of this study are available upon request.

## Supplementary Materials

The following are available online at www.mdpi.com/article/10.3390/plants10102128/s1, Figure S1: Climatological conditions and general crop phenology. Figure S2: Fresh and Dry weight of 25 panicles. Figure S3: Correlogram of variables measured. Figure S4: Pictures of seeds of the different cultivars harvested and analyzed in this study. Table S1: Direct effects of predictor variables of the first-, second-, third-, and fourth-order on germination rate, tolerance, and variance inflation factor of the path analysis. Table S2: Direct effects of predictor variables of the first-, second-, third-, and fourth-order on germination rate, tolerance, and variance inflation factor of the path analysis.
